# Maternal Hepatitis B Infection Burden, Comorbidity and Pregnancy Outcome in a Low-Income Population on the Myanmar-Thailand Border: A Retrospective Cohort Study

**DOI:** 10.1155/2019/8435019

**Published:** 2019-02-25

**Authors:** Marieke Bierhoff, Chaisiri Angkurawaranon, Aung Myat Min, Mary Ellen Gilder, Nay Win Tun, Arunrot Keereevijitt, Aye Kyi Win, Elsi Win, Verena Ilona Carrara, Tobias Brummaier, Cindy S. Chu, Laurence Thielemans, Kanlaya Sriprawat, Borimas Hanboonkunupakarn, Marcus Rijken, François Nosten, Michele van Vugt, Rose McGready

**Affiliations:** ^1^Shoklo Malaria Research Unit, Mahidol-Oxford Tropical Medicine Research Unit, Mahidol University, Mae Sot 63110, Thailand; ^2^Division of Infectious Diseases, Academic Medical Center, University of Amsterdam, Amsterdam, Netherlands; ^3^Department of Family Medicine, Faculty of Medicine, Chiang Mai University, Chiang Mai 50200, Thailand; ^4^Department of Medicine, Swiss Tropical and Public Health Institute, Basel, Switzerland; ^5^Faculty of Medicine, University of Basel, Basel, Switzerland; ^6^Neonatology-Pediatrics, Cliniques Universitaires de Bruxelles-Hôspital Erasme, Université Libre de Bruxelles, Brussels, Belgium; ^7^Mahidol-Oxford Tropical Medicine Research Unit, Mahidol University, Bangkok 10400, Thailand; ^8^Utrecht University Medical Centre, Utrecht, Netherlands, and Julius Centre Global Health, Utrecht 3584 CX, Netherlands; ^9^Centre for Tropical Medicine and Global Health, Nuffield Department of Medicine Research Building, University of Oxford, Oxford OX3 7FZ, UK

## Abstract

**Objectives:**

Hepatitis B virus (HBV) was believed to have minimal impact on pregnancy outcomes apart from the risk of perinatal transmission. In more recent years, there have been reports of adverse associations, most consistently preterm birth (PTB), but this is in the context of high rates of caesarean section. The aim of this study was to explore the association of HBV on pregnancy outcomes in marginalized, low-income populations on the Myanmar-Thailand border.

**Methods:**

HBsAg positive (+) point of care rapid detection tests results were confirmed by immunoassays. Women with a confirmed HBsAg status, HIV- and syphilis-negative at first antenatal care screening, singleton fetus and known pregnancy outcome (Aug-2012 to Dec-2016) were included. Logistic regression analysis was used to evaluate associations between HBV group (controls HBsAg negative, HBsAg+/HBeAg-, or HBsAg+/HBeAg+) and pregnancy outcome and comorbidity.

**Results:**

Most women were tested, 15,046/15,114 (99.6%) for HBV. The inclusion criteria were not met for 4,089/15,046 (27.2%) women due mainly to unavailability of pregnancy outcome and nonconfirmation of HBsAg+. In evaluable women 687/11,025 (6.2%) were HBsAg+, with 476/11,025 (4.3%) HBsAg+/HBeAg- and 211/11,025 (1.9%) were HBsAg+/HBeAg+. The caesarean section rate was low at 522/8,963 (5.8%). No significant associations were observed between pregnancy comorbidities or adverse pregnancy outcomes and HBV status.

**Conclusions:**

The results highlight the disease burden of HBV in women on the Myanmar-Thailand border and support original reports of a lack of significant associations with HBsAg+ irrespective of HBeAg status, for comorbidity, and pregnancy outcomes in deliveries supervised by skilled birth attendants.

## 1. Introduction

Hepatitis B virus (HBV) infection is hyperendemic in Southeast Asia. It is assumed that about 75-80% of the estimated 240 million HBV carriers globally live in this region [1]. In endemic areas in Southeast Asia and Africa, where the most significant route of transmission is from mother to child (MTCT) or from child to child, up to 90% of infected persons have a chronic course [2, 3]. In mothers who are HBeAg positive (+) and at highest risk of transmitting HBV, Hepatitis B immunoglobulins (HBIG) should be routinely provided if women give birth or in the case of homebirth the infant should be presented to a clinic where this specialized vaccination is available before 72 hours of life [4, 5]. However, this prophylactic regimen is often not given in low-income countries (LIC) because of cost, complexity of production, and need for a reliable cold chain [6]. Health care systems in LIC struggle to respond to the significant burden of communicable infections in pregnancy and routine HBV testing is not always available [7].

For many years it has been thought that maternal HBV infection had no influence on pregnancy outcomes [8], but published evidence particularly from the last 5 years suggests there may be an impact of HBsAg+ irrespective of HBeAg status on preterm birth, among other outcomes [9–14]. Preterm birth (PTB) has been highlighted as a major finding in three [10, 13, 14] of these publications. While there is theoretical evidence to support an increased risk of PTB from chronic liver disease due to increased cytokine production from inflammation [15], the PTB data remains largely observational and at risk of bias. Sources of bias could include inability to control use of other (social) drugs, other (subclinical) infections, e.g., Hepatitis C virus, poor gestational age assessment, and obstetrician preference for caesarean section [16], a known iatrogenic risk factor for PTB. The leading countries contributing to data on pregnancy outcome according to HBV status are the USA and Europe who have low HBV prevalence and China with a moderate to high HBV prevalence; USA and China have high rates of caesarean section [17]. Controlling the indication for caesarean section in data extraction for systematic review and meta-analysis can be problematic as country policy and local hospital practice may not confer. HBsAg+, irrespective of HBeAg status, was associated with miscarriage in one study [11] where more than 20,000 women were registered since the first trimester. Other studies have not found associations between HBsAg+ (irrespective of HBeAg status) and pregnancy outcomes and pregnancy morbidity including premature rupture of membranes, preeclampsia, gestational diabetes mellitus (GDM), increased risk of prematurity, lower birth weight, small- or large for gestational age or antepartum hemorrhage [10–12].

A high burden of HBV (8.3%) infection in refugees and migrant pregnant women on the Myanmar-Thailand border as well as factors associated with infection (age over 25 years and Karen heritage) for the period of August 2012 and April 2014 has been reported [18]. In this retrospective study we aimed to examine the association of HBV infection with coinfection, pregnancy morbidity, and pregnancy outcomes, including caesarean section, by expanding upon the previous cohort presenting data from August 2012 to December 2016, and previously unpublished data on pregnancy outcomes.

## 2. Methods

### 2.1. Study Design

This is a retrospective cohort study of refugee and migrant women registered at antenatal clinics on the Myanmar-Thailand border and screened for HBV.

### 2.2. Setting

Shoklo Malaria Research Unit (SMRU) provides humanitarian health care for marginalized populations in north western Thailand on the border with Myanmar, with a focus on maternal and child health. At the time of data collection, antenatal care (ANC) and birthing services were available at three sites: two migrant sites, Mawker Thai (MKT) and Wang-Pha (WPA), and one refugee site, Maela (MLA) camp. Three decades of maternal and child health care on the Thailand-Myanmar border have resulted in significant trust in the services provided by SMRU among the local population. Karen/Kayin state in eastern Myanmar is a difficult geographical setting—roads become impassable in the rainy season making it impossible to reach clinics [19]. Attendance at ANC is voluntary and uptake is high. In the migrant population attendance and delivery have continued to increase, in part because access in rural areas to maternal and child health services provided in Karen and Burmese language is limited, and health care in Myanmar and Thailand is associated with significant household expenditure [20, 21]. In rural eastern Myanmar up to 3/4 women give birth at home [22].

Antiretroviral treatment of HBV in pregnancy is not supported by the government in Myanmar or in Thailand in pregnancy, and while it could be obtained from out of pocket expenses this would be beyond the means of this marginalized population. Trust in government services in Myanmar is low with significant out-of-pocket expenditure, so most poor rural populations avoid seeking care at tertiary hospitals [23].

### 2.3. Participants

Participants were refugee and migrant women registered at SMRU ANC on the Thailand Myanmar.

### 2.4. Routine ANC and Birth Procedures

Routine counseling and screening commenced in MKT on 08-Aug-2012, in WPA on16-Aug-2012, and in MLA on 28-Aug-2012. The HBV test was offered at the first ANC visit along with screening for other infections, Human Immunodeficiency Virus (HIV), syphilis, common tropical infections, malaria, and soil transmitted helminths [22]. Women that test positive for HBV infection do not get treated with antiviral therapy.

Malaria smears were done every two weeks in the migrant setting and for the first three visits for refugees and at any time if the woman complained of malaria symptoms, had a fever, or had preterm labor. All women routinely received prophylactic supplements including ferrous sulphate 200 mg daily, folic acid 5mg per week, and vitamin B1 100 mg daily. Haematocrit (HCT) was measured at each visit, with most women checked at least four times by delivery. Any HCT value < 30% was defined as anemia in pregnancy. General maternal examination was performed according to a set schedule, including weight, blood pressure, and fundal height. In women with hypertension (BP ≥140/90 mmHg), proteinuria was tested at least twice 6 hours apart to differentiate pregnancy induced hypertension from preeclampsia. Eclampsia was defined by hypertension and convulsions. Low (<18.5 kg/m2), normal (18.5-23 kg/m2), and high (≥23 kg/m2) BMI were defined using WHO standards for Asian populations [24]. All women were provided with an ultrasound assessment at the first ANC visit, determining the number of fetus as well as enabling the estimation of gestational age (EGA). Gestational Diabetes Mellitus (GDM) screening with 75g oral glucose tolerance test was based on risk factors developed for this population [25]. Miscarriage included loss of the pregnancy before 28 weeks. Preterm birth (PTB) was delivery before 37 weeks' gestational age and size for gestational age was determined using international standards [26]. Women were encouraged to deliver at SMRU clinics with skilled birth attendants [27]. Women requiring caesarean section were referred out to the nearest Thai Public Hospital; the majority of these are for emergencies only or when booked, for clearly documented pathologies such as placenta praevia. Caesarean section by maternal request or without a medical or obstetric indication is not available. Every newborn had a physical examination conducted by trained clinical staff with results recorded on a standard reporting form [28].

### 2.5. Laboratory Methods

Initial screening for HBV was performed using a rapid diagnostic test (RDT, Pacific Biotech, Thailand) with a reported sensitivity of 100% (63.1-100%) and specificity of 100% (98.9-100%) [29]. The high sensitivity and specificity are not consistent with the false positive rate of 3.1% (95% CI 1.7- 5.4) in pregnant women previously reported by SMRU [18]. Serum from the women with RDT HBsAg-positive was sent for verification using the* HBsAg electrochemiluminescence immunoassay *(ECLIA) on Cobas e immunoassay analyzer (Roche Diagnostics, Indianapolis, USA) and from June 2014 with the Chemiluminescence immunoassay (CLIA) method (Unicel DxI1800, Beckman Coulter). This was conducted independently at the local tertiary referral provincial hospital in Thailand (the assay has been CE marked according to Directive 98/79/EC). The* HBeAg electrochemiluminescence immunoassay *was also carried out in the external laboratory on Cobas e immunoassay analyzer (Roche Diagnostics, USA).

HIV was screened using a one-step Anti-HIV (1&2) Tri-Line Test and confirmed by a second test if the first test was reported as reactive, syphilis by VDRL and TPHA, malaria by microscopy of Giemsa stained thick blood smear, and declared negative if no parasites were detected in 200 high powered fields, and soil transmitted helminths from fecal samples were processed the same day using the formalin concentration method for detection of helminth eggs [30].

### 2.6. Inclusion and Exclusion Criteria

**Figure 1:**
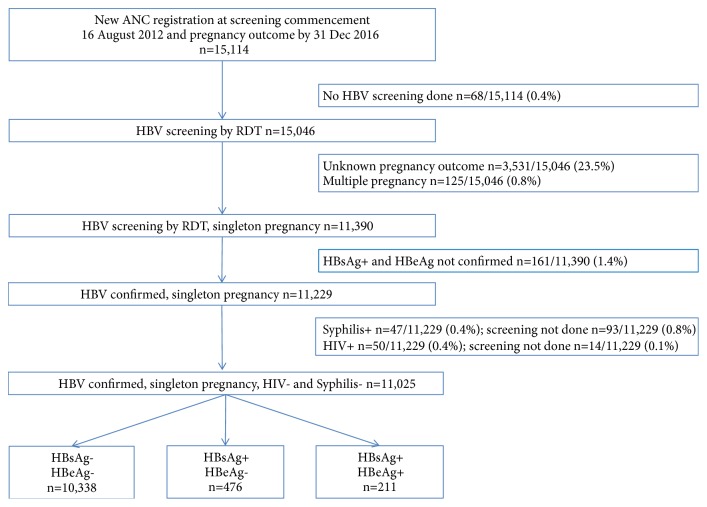
Inclusions and exclusions for the analyses.

All women who attended the ANC of SMRU starting 16 August 2012 and had a delivery outcome by 31 December 2016 were screened for inclusion in this analysis (Figure 1). Women with a singleton fetus, confirmation of positive HBsAg RDT, who screened negative for HIV and syphilis, and had a known outcome of pregnancy were included. In this highly mobile population approximately 20% of women register to antenatal care but move, usually for work, before the outcome of pregnancy is known. Low burden of HIV 17/3,599 (0.47%, 95% CI 0.30-0.76) and syphilis 14/3,592 (0.39%, 95% CI 0.23-0.65) has previously been reported in pregnant women in this area [31]. There was no data on Hepatitis C infections or other sexual transmitted diseases. Rates of multiple pregnancies in this area are low at approximately 1% and there is no assisted fertility treatment.

### 2.7. Statistical Analysis

Data were analyzed using SPSS version 23. Demographic characteristics of the three groups (controls, HBsAg+/HBeAg- and HBsAg+/HBeAg+) were compared using the Chi-squared test for categorical variables and the Student's* t*-test or Mann–Whitney-U test for continuous data.

The Pearson Chi-squared test was used to examine the association of HBV infection and comorbidity and HBV infection and pregnancy outcome: computing crude odds ratio (OR) with 95% confidence intervals (CIs). Logistic regression models were used to study the association between HBV infection and comorbidity and HBV infection and pregnancy outcomes. Bonferroni correction for multiple testing was performed for p<0.05. The variables were checked for confounding and effect modification for the stated pregnancy outcomes.

Reported demographic variables included categorical: gravidity, parity, BMI, height; dichotomous: age younger than 25 years, Karen ethnicity, migrant status, literacy, smoking, attendance of ANC in the first trimester, underweight anytime in pregnancy and in first trimester (BMI as a proxy for prepregnancy weight, and in women with gravidity >1 a previous history of miscarriage and PTB). Coinfections during pregnancy included malaria, nonmalaria febrile illnesses during pregnancy (temperature of >37.5 degrees Celsius), and soil transmitted helminths (including Hookworm as* Necator americanus *and* Ancylostoma duodenale* ova which cannot be differentiated by microscopy,* Ascaris lumbricoides* (roundworm) or* Trichuris (T.) trichiura* (whipworm)). Pregnancy related morbidity included anemia, pregnancy induced hypertension, preeclampsia and eclampsia and GDM. Available pregnancy outcomes included maternal death, miscarriage, delivery, gestational age, PTB, stillbirth, congenital abnormality, birthweight, small for gestational age (SGA), large for gestation age (LGA), delivery by caesarean section, and neonatal death. For analysis related to birth weight, only liveborn, congenitally normal infants with a valid birth weight within 72 hours after birth were included.

### 2.8. Ethics

This study was approved by the local Tak Community Advisory Board (20171028-TCAB-13), Oxford Tropical Research Ethics Committee (OXTREC 28-09), and the Chiang Mai University Research Ethics Committee (FAM-2560-05195). The patient data were anonymized before accessing and entering them for this study.

## 3. Results

The majority of women who presented to SMRU ANC between August 2012 and December 2016, 15,046/15,115 (99.6%), were tested for HBV infection (Figure 1). The inclusion criteria were not met for 4,089/15,046 (27.2%) of women with the two main exclusions being unavailability of pregnancy outcome (3,531/4,089 women, 86.4%), an expected result in this highly mobile population, and nonconfirmation of HBsAg+ RDT (161/4,089, 3.9%).

There were differences in the baseline characteristics of women who were included and excluded (n=4,089) (S2 Table). Unavailability of pregnancy outcomes occurred in each trimester as seen by trimester of last antenatal visit (S2 Table) and included women who left the catchment area for work or to deliver with more family support in their home town or from a pregnancy outcome elsewhere. It is more difficult for migrant women and visitors to the refugee camp, especially those whose residence is in Myanmar, to cross the border (which is a river) to reach the birthing services, than for women residing at the camp who are within walking distance from the clinic (S2 Table). No significant difference in the proportion of HBsAg+ was observed for included 687/11,025 and excluded cases 250/3,802 (6.6%), p=0.464 (S2 Table).

The only differences in the comparisons of demographic (S3 table) and delivery characteristics (S4 Table) for HBsAg+ confirmed (included women) and HBsAg+ unconfirmed (excluded women) were for status where the proportion of migrants was higher and first trimester attendees lower, in excluded women. For HBeAg+ women who were included and excluded significant differences were observed in the mean age of excluded women, which was slightly older, and ethnicity, as excluded women were less likely to be Karen (S5 Table).

Among the 11,025 women included in the final analyzes, the proportion of HBsAg+ was 687/11,025 (6.2%, 95% CI 5.7-6.7). There were 476/11,025 (4.3%) of the women grouped as HBsAg+/HBeAg- and 211/11,025 (1.9%) as HBsAg+/HBeAg+.

### 3.1. Demographic Characteristics

The characteristics of eligible pregnant women according to HBV infection status are presented in Table 1. Women with HBsAg+/HBeAg- and HBsAg+/HBeAg+ differed from controls and from each other. A significantly higher proportion of women with HBsAg+/HBeAg- were of older age, whereas those with HBsAg+/HBeAg+ were of younger age. Given these significant differences in age, variations in obstetric (gravidity, parity, and primigravida) and BMI characteristics (overweight) are not unexpected (Table 1).

A higher proportion of women with Karen heritage had HBV infection, but a higher proportion of refugees were HBsAg+/HBeAg- while migrants were HBsAg+/HBeAg+ (Table 1). There was no significant difference between the groups for literacy, the proportion of first ANC visits in first trimester, maternal height, and in multigravid women, a history of previous abortion, and PTB.

### 3.2. Pregnancy Comorbidity

The proportion of coinfections (malaria, soil transmitted helminths) detected by screening and pregnancy related morbidity that occurred over the course of gestation (anemia, pregnancy induced hypertension, eclampsia or preeclampsia, or GDM were similar between groups) were similar between groups (Table 2). Nonmalaria febrile illness was lower in the HBsAg+/HBeAg- compared to the control group (OR 0.53, 95% CI 0.31-0.89, p=0.017).

For individual species of soil transmitted helminths, the proportion of* Hookworm *and* Trichuris Trichiura* were similar by HBV status but there was a higher, nonsignificant proportion of* Ascaris lumbricoides* infection in women with HBsAg+/HBeAg+: 36/177 (20.3%) compared to controls 1,245/7,8201 (15.2%) OR 1.43 (0.98-2.07), p= 0.061.

In a logistic regression model, risk factors associated with HBsAg+/HBeAg+ compared to controls included* Ascaris lumbricoides* infection aOR 1.59 (1.08-2.35), p=0.020; age less than 25 years aOR 2.64 (1.91-3.65), p<0.001; migrant status aOR 1.57 (1.12-2.19); p=0.008, and Karen heritage aOR 1.79 (1.25-2.56), p=002.

### 3.3. Pregnancy Outcomes

The proportion of maternal mortality was highest in the control group compared to the HBsAg+/HBeAg- and HBsAg+/HBeAg+ groups (Table 3). In this cohort HBV was not associated with an increased risk of miscarriage, PTB, stillbirth, congenital abnormality, or neonatal death, and HBV did not have a negative effect on mean birthweight or SGA.

The proportion of women who delivered by caesarean section was low: 522/8,963 (5.8%). There was a higher proportion of women in the HBsAg+/HBeAg- group who delivered by caesarean section (34/379 (9.0%) compared to controls 480/8,417 (5.7%) with an OR 1.61 (95%CI 1.13-2.30), p= 0.008) on univariate analysis, but, with Bonferroni correction for multiple testing, the difference was no longer statistically significant, p=0.064 (Table 3).

## 4. Discussion

This review including more than 11,000 women from a low-income setting does not support an increased risk of adverse birth outcomes for those women with HBsAg+ irrespective of HBeAg status compared to controls when pregnancy outcomes were supervised by skilled birth attendants. In multigravid women who attended ANC from the first trimester, there was no increased risk of miscarriage with HBV, which differs from recent previous reports [11, 32]. Pregnancy associated morbidity (GDM, eclampsia and preeclampsia) and outcomes of stillbirth and PTB were also not significantly different by HBV status in this cohort [33, 34]. Careful interpretation of obstetric outcomes is required due to the linear association of HBV infection with age and with age and gravidity.

The absence of adverse pregnancy outcomes observed here and reported elsewhere could be related to the chronic character of HBV infection [35]. In this population we expect that most of the women will have acquired their HBV infection preconceptually or during childhood. The earlier publications on HBV and adverse pregnancy outcomes emphasized poor outcomes with acquisition of acute HBV infection in late pregnancy [36, 37]. For the women in this population with an HBsAg+ and especially HBeAg+ status, the dominant risk of HBV infection in pregnancy is the unmeasured risk of transmission of infection from mother to child. This is a problem in LIC with high HBV burden as the expanded program of immunization can be suboptimal, HBIG is usually not available, and women often labor without skilled birth attendance at home [6]. There is currently a shift towards consideration of alternative strategies to relying on vaccination and HBIG to interrupt transmission, such as antiretroviral therapy with tenofovir [6].

### 4.1. Hepatitis B Virus Burden

Although HBV is recognized as being highly prevalent in Southeast Asia, in this sample of pregnant women there was a proportion of HBsAg+ of 6.2% (95%CI 5.7-6.7) with almost one-third who were also HBeAg+ (30.7%; 95%CI 29.8-31.6). Comparison of HBV prevalence between different countries revealed that our estimates of HBV in Thailand are lower than those reported for other low and middle income countries. An Asian study among 3009 samples reported a prevalence of HBsAg positivity of 9.4% with 121 Cambodians (10.8%), 54 Laotians (6.9%), and 107 from Myanmar (9.7%) [38]. In high income countries the prevalence is much lower than that reported for Asia; for instance, in northern America and western Europe, the prevalence is between 1-2%. The difference in prevalence with high income countries could be due to a better implementation of the vaccination program on top of the previous immunization of high risk groups and lower population prevalence of HBV before vaccination became available [39]. This is further supported by the overall decrease in HBV prevalence from 1990 to 2005 in younger age groups [40, 41].

### 4.2. Pregnancy Coinfections

There was no significant association between HBV and malaria, but the overall proportion of women infected with malaria in this cohort was low. This low level of malaria is expected given very active control programs on both sides of the border in the study area [42, 43]. There was a nonsignificant higher proportion of* A. Lumbricoides* in women with HBsAg+/HBeAg+. It is possible that HBsAg+/HBeAg+ contributes to a reduced immune response to helminthic infection, or* vice versa* [44–46]. Integration of screening and deworming should be considered in studies considering ARV PMTCT of HBV in areas with high rates of soil transmitted helminths in the population [6, 44].

### 4.3. Limitations

The major limitation of this analysis is the 27.2% of women who booked at ANC but had unavailable pregnancy outcome or no confirmation of the positive point of care rapid test result. Reassuringly there were no important differences between included and excluded women and in demographic and delivery outcomes of HBsAg confirmed and unconfirmed women (S2–S4 Tables) reducing the risk of bias in reported outcomes such as miscarriage and PTB. Data needs to be considered in context and on the Thailand-Myanmar border it is very unlikely that these marginalized women were able to selectively choose more sophisticated care for their HBV status or meet the out of pocket expenditure for basic mother and child health care in Myanmar [23].

GDM screening employed a risk-factor based approach due to cost constraints, so the prevalence is likely an underestimate. Only 4/5 of women provided stool samples, so cautious interpretation of helminth data is required. Finally only indirect markers (HBsAg and HBeAg) of HBV infection were available.

In this as in other cohorts, the precise indication for caesarean section was not available, potentially leading to bias in pregnancy outcomes [17]. However, the indications in this setting for caesarean section are for emergencies in >90% of cases and booked cases have a significant pregnancy related complication, such as placenta previa. The associations observed in this cohort are very similar to older reports on HBV when caesarean section rates were much lower than current reports [47].

## 5. Conclusions

Overall, the results highlight the disease burden of HBV in women on the Myanmar-Thailand border. The mobility of these populations is significant as one in four women did not have their final pregnancy outcome at the clinic where they registered and followed antenatal care. In births supervised by skilled birth attendants, there were no significant associations between HBsAg+ irrespective of HBeAg status for comorbidity or for pregnancy outcomes.

## Figures and Tables

**Table 1:** Baseline characteristics of the study population, comparing the three investigated groups with an overall p value.

	**All women**	**Controls** ^**∗**^	**HBsAg+/HBeAg- **	**HBsAg+/HBeAg+**	**P value** ^1^
	n=11,025	n=10,338	n=476	n=211	
**Age yrs, mean ± SD (min-max)**	26 ± 7 (13-50)	26 ± 7 (13-50)	29 ± 7 (15-47)	23 ± 6 (15-45)	<0.001

**Age<25 years**	5,060 (45.9)	4,782/10,338 (46.3)	134/476 (28.2)	144/211 (68.2)	<0.001

**Gravidity median [IQR] **	2 [1-19]	2 [1-4]	3 [2-5]	2 [1-3]	<0.001

**Parity median [IQR] **	1 [0-15]	1 [0-2]	2 [1-3]	1 [0-2]	<0.001

**Primigravida**	3,522 (31.9)	3,349/10,338 (32.4)	90/476 (18.9)	83/211 (39.3)	<0.001

**Karen ethnicity**	7,162 (65.0)	6,667/10,338 (64.5)	341/476 (71.6)	154/211 (73.0)	0.002

**Status Migrant (not refugee)**	6,452 (58.5)	6,063/10,338 (58.6)	249/476 (52.3)	140/211 (66.4)	0.002

**Literate**	6,728 (61.0)	6,330/10,338 (61.2)	274/476 (57.6)	124/211 (58.8)	0.219

**Smoker**	1,440 (13.1)	1,338/10,338 (12.9)	80/476 (16.8)	22/211 (10.4)	0.026

**First ANC visit in 1st trimester**	4,479 (40.6)	4,189/10,337 (40.5)	206/476 (43.3)	83/211 (39.3)	0.479

**EGA in weeks at first ANC median [IQR]**	17 [10-26]	17 [10-26]	16 [10-24]	18 [11-25]	0.181

**Underweight** ^**2**^ **, (BMI**<**18.5 kg/m**^**2**^**)**	724/4,463 (16.2)	690/4,173 (16.5)	24/206 (11.7)	9/83 (10.8)	0.174

**Overweight** ^**3**^ **, (BMI**≥**23 kg/m**^**2**^**)**	1,236/4,463 (27.7)	1,142/4,173 (27.4)	75/206 (36.4)	19/83 (22.9)	<0.001

**Height (cm) mean (min-max)**	151 ± 5 (130-191)	151 ± 5 (130-191)	152 ± 5 (134-174)	152 ± 6 (137-174)	0.937

**Previous miscarriage (Gravidity **>**1)**	2,321/7,503 (30.9)	2,171/6,989 (31.1)	114/386 (29.5)	36/211 (28.1)	0.108

**Previous PTB (Gravidity**>**1)**	589/7,503 (7.9)	541/6,989 (7.7)	33/386 (8.5)	15/128 (11.7)	0.144

^*∗*^HBsAg negative.

Data are n (%), mean ± standard deviation (SD) (min-max); median inter-quartile range [IQR] (min-max). Abbreviations: ANC, antenatal clinic, BMI, body mass index, PTB, preterm birth.

Missing data: BMI missing for 38 women for all women and 16 with first ANC in trimester one. ^1^P value: proportions compared by Chi-square test, means by Students t-test; median by the Mann-Whitney U test, ^2^BMI regardless of trimester at first ANC visit; underweight - BMI <18.5 mg/kg2 compared to normal weight - BMI 18.5 to <23.5 mg/kg2.

^3^BMI if trimester at first ANC was trimester one, as a proxy for prepregnancy weight; over weight BMI ≥23.5 mg/kg2 compared to normal weight.

**Table 2:** Association between HBV infection and comorbidity during pregnancy.

	**Controls** ^**∗**^	**HBsAg+/HBeAg- **	**HBsAg+/HBeAg+**	**OR (95**%**CI), P value**	
	n=10,338	n=476	n=211	control *vs.* HBsAg+/HBeAg-	control *vs.* HBsAg+/HBeAg+
**Malaria**	273/10,338 (2.6)	6/476 (1.3)	8/211 (3.8)	0.47 (0.21-1.06), 0.070	1.45 (0.71-2.98), 0.307

**Febrile illness pregnancy**	599/10,338 (5.8)	15/476 (3.2)	11/211 (5.2)	0.53 (0.31-0.89), 0.119	0.89 (0.49-1.65), 0.721

**Soil transmitted helminth**	1957/8,201 (23.9)	86/416 (20.7)	47/177 (26.6)	0.83 (0.65-1.06), 0.136	1.15 (0.82-1.62), 0.407

* Hookworm*	563/8,201 (6.9)	20/416 (4.8)	9/177 (5.1)	0.73 (0.46-1.15), 0.169	0.77 (0.39-1.51), 0.447

* Ascaris Lumbricoides*	1,245/8,201 (15.2)	55/416 (13.2)	36/177 (20.3)	0.85 (0.64-1.14), 0.276	1.43 (0.98-2.07), 0.061

* Trichuris Trichiura*	377/8,201 (4.6)	13/416(3.1)	10/177 (5.6)	0.67 (0.38-1.17), 0.162	1.23 (0.65-2.37), 0.510

**Anemia in pregnancy**	1477/10,325 (14.3)	58/475 (12.2)	35/211 (16.6)	0.83 (0.63-1.10), 0.202	1.19 (0.83-1.72), 0.350

**Pregnancy induced hypertension** ^1^	834/10,338 (8.1)	39476 (8.2)	16/211 (7.6)	1.02 (0.73-1.42), 0.921	0.94 (0.56-1.56), 0.798

**Eclampsia or Preeclampsia** ^1^	1,000/10,338 (9.7)	44/476 (9.2)	21/211 (10.0)	0.95 (0.69-1.31), 0.756	1.03 (0.66-1.63), 0.890

**Diabetes in pregnancy** ^2^	539/7,932 (6.8)	22/409 (5.4)	5/171(2.9)	0.78 (0.50-1.21), 0.266	0.41 (0.17-1.01), 0.053

^*∗*^HBsAg negative.

Abbreviations: SD, standard deviation, GDM, gestational diabetes mellitus.

Missing values: anemia (n=14), soil transmitted helminths (n=2,231) with 2137,60,34 in each group, respectively.

^1^Pregnancy inducted hypertension and eclampsia or preeclampsia are mutually exclusive: final hypertension diagnosis was the most severe form observed in pregnancy.

^2^Gestational diabetes screening (75g OGTT) commenced in August, 2013, hence having lower denominator; cases included 12 women in control group with diabetes at first ANC.

**Table 3:** Association between HBV infection and pregnancy outcomes.

	**Controls** ^**∗**^	**HBsAg+/HBeAg- **	**HBsAg+/HBeAg+**	**Overall P value**	**Crude OR (95**%** CI), P value**	
	n=10,338	n=476	n=211		*control vs. HBsAg+/HBeAg- *	*control vs. HBsAg+/HBeAg+*
**Maternal deaths**	6/10,388 (0.6%)	2/476 (0.4%)	0	too few cases	too few cases	too few cases

**Miscarriage (1st ANC Trimester 1)**	832/4,190 (19.9%)	48/206 (23.3%)	15/83 (18.1%)	0.435	1.23 (0.88-1.71), 0.226	0.94 (0.71-1.25), 0.690

**Delivered **	n=9.326	n=419	n=188			

**Gestation, weeks mean ± SD (min-max)**	39.0 ± 1.7 (28.0-44.2)	39.1 ± 1.7 (28.1-43.2)	39.1 ± 1.3 (33.3-41.5)		p= 0.228	p= 0.350

**Preterm birth, (<37 wks)**	671/9,326 (7.2%)	26/419 (6.2%)	8/188 (4.3%)	0.230	1.17 (0.78-1.76), 0.442	1.32 (0.93-1.89), 0.126

**Stillbirth**	82/9,323 (0.9%)	4/419 (1.0%)	1/188 (0.5%)	0.898	1.09 (0.40-2.98), 0.872	0.60 (0.08-4.35), 0.616

**Congenital abnormality**	193/9,322 (2.1%)	7/418 (1.7%)	2/188 (1.1%)	0.487	0.81 (0.38-1.72), 0.577	0.71 (0.35-1.44), 0.344

**If liveborn, normal singleton**	n=9,058	n=408	n=185			

**Weighed in 72 hrs of birth**	8,420/9,065 (93.0%)	380/408 (93.1%)	167/185 (90.3%)	0.317	0.97 (0.66-1.44), 0.896	1.19 (0.93-1.53), 0.159

**Birthweight, grams** **mean ± SD (min-max)**	2,998 ± 452 (700-5,350)	3,044 ± 432 (1,000-4,340)	3,012 ± 417 (1,840-4,140)		p= 0.046	p=0.670

**Small for gestational age**	1,566/8,395 (18.7%)	54/378 (14.3%)	30/166 (18.1%)	0.100	0.73 (0.54-0.97), 0.264	0.98 (0.80-1.20), 0.849

**Large for gestational age**	196/8,395 (2.3%)	8/378 (2.1%)	4/166 (2.4%)		1.17 (0.91-1.50), 0.228	0.93 (0.44-1.98), 0.851

**Delivered by Caesarean section**	480/8,417 (5.7%)	34/379 (9.0%)	8/167 (4.8%)	0.329	1.61 (1.13-2.30), 0.064	0.95 (0.50-1.81), 0.876

**Neonatal death**	44/6,533 (0.7%)	3/324 (0.9%)	0/137	too few cases	too few cases	too few cases

^*∗*^HBsAg negative.

Data are n (%) unless otherwise stated.

Abbreviations: SD, standard deviation, GDM, gestational diabetes mellitus.

Values reported as mean ± SD were compared with t test, proportions with Chi-square test, median with Mann-Whitney U test: with Bonferroni correction for multiple testing if p<0.05.

## Data Availability

The data used to support the findings of this study may be released upon application to the Data Access Committee at Mahidol-Oxford Tropical Medicine Research Unit (MORU), who can be contacted at http://www.tropmedres.ac/data-sharing.

## Supplementary Materials

Supplementary Materials
* Table S1. Reported associations of HBV and pregnancy outcome from case control, systematic reviews, meta-analysis, and large (>20,000) population based cohorts from the last 5 years*.* Abbreviations (alphabetic order).* Antepartum hemorrhage: APH, adjusted Odds Ratio: aOR, birth weight: BW, Caesarean Section: CS, Cohort (large >20,000 population based), Congenital abnormality: CA, Eclampsia: EC, intrahepatic cholestasis of pregnancy: ICP, large for gestational age: LGA, low birth weight: LBW, meta-analysis: MA; Neonatal Jaundice: NJ, Odds Ratio: OR, pooled Odds Ratio: pOR, pooled Risk Ratio: pRR, Preeclampsia: Pre-EC, Pregnancy induced hypertension (or gestational hypertension): PIH, Preterm birth: PTB, Preterm rupture of membranous: PROM, Small for gestational age: SGA, Stillbirth: SB, Systematic Review: SR.* Table S2. Characteristics of all women screened for hepatitis B virus*. Data are n (%) unless otherwise stated, ^1^p value of 0.05 was considered statistically significant.* Table S3. Baseline characteristics of the included and excluded pregnant women with HBsAg confirmed and unconfirmed status*. Data are n (%), mean ± standard deviation (SD) (min-max); median interquartile range [IQR] (min-max).* Abbreviations.* ANC: antenatal clinic, BMI: body mass index, PTB: preterm birth. ^1^p value of 0.05 was considered statistically significant, and ^2^BMI if trimester at first ANC was trimester one, as a proxy for prepregnancy weight.* Table S4. Pregnancy outcomes in HBsAg confirmed and HBsAg+ but unconfirmed women*. Data are n (%) unless otherwise stated. ^1^p value of 0.05 was considered statistically significant.* Table S5. Baseline characteristics of the included and excluded pregnant women with HBeAg confirmed status*. Data are n (%), mean ± standard deviation (SD) (min-max); median interquartile range [IQR] (min-max).* Abbreviations.* ANC: antenatal clinic, BMI: body mass index, PTB: preterm birth. ^1^p value of 0.05 was considered statistically significant, and ^2^BMI if trimester at first ANC was trimester one, as a proxy for prepregnancy weight.
