# Supplementary material for: Maternal Hepatitis B Infection Burden, Comorbidity and Pregnancy Outcome in a Low-Income Population on the Myanmar-Thailand Border: A Retrospective Cohort Study
Source: J Pregnancy. 2019 Feb 25;2019:8435019. doi: 10.1155/2019/8435019 (PMC6413357; doi:10.1155/2019/8435019)
Supplement: Supplementary Materials — Table S1. Reported associations of HBV and pregnancy outcome from case control, systematic reviews, meta-analysis, and large (>20,000) population based cohorts from the last 5 years. Abbreviations (alphabetic order). Antepartum hemorrhage: APH, adjusted Odds Ratio: aOR, birth weight: BW, Caesarean Section: CS, Cohort (large >20,000 population based), Congenital abnormality: CA, Eclampsia: EC, intrahepatic cholestasis of pregnancy: ICP, large for gestational age: LGA, low birth weight: LBW, meta-analysis: MA; Neonatal Jaundice: NJ, Odds Ratio: OR, pooled Odds Ratio: pOR, pooled Risk Ratio: pRR, Preeclampsia: Pre-EC, Pregnancy induced hypertension (or gestational hypertension): PIH, Preterm birth: PTB, Preterm rupture of membranous: PROM, Small for gestational age: SGA, Stillbirth: SB, Systematic Review: SR. Table S2. Characteristics of all women screened for hepatitis B virus. Data are n (%) unless otherwise stated, 1p value of 0.05 was considered statistically significant. Table S3. Baseline characteristics of the included and excluded pregnant women with HBsAg confirmed and unconfirmed status. Data are n (%), mean ± standard deviation (SD) (min-max); median interquartile range [IQR] (min-max). Abbreviations. ANC: antenatal clinic, BMI: body mass index, PTB: preterm birth. 1p value of 0.05 was considered statistically significant, and 2BMI if trimester at first ANC was trimester one, as a proxy for prepregnancy weight. Table S4. Pregnancy outcomes in HBsAg confirmed and HBsAg+ but unconfirmed women. Data are n (%) unless otherwise stated. 1p value of 0.05 was considered statistically significant. Table S5. Baseline characteristics of the included and excluded pregnant women with HBeAg confirmed status. Data are n (%), mean ± standard deviation (SD) (min-max); median interquartile range [IQR] (min-max). Abbreviations. ANC: antenatal clinic, BMI: body mass index, PTB: preterm birth. 1p value of 0.05 was considered statistically significant, and 2BMI if trimester at fi [file 8435019.f1.docx]

**S1 Table: Reported associations of HBV and pregnancy outcome from case control, systematic reviews, meta-analysis and large (>20,000) population based cohorts from the last 5 years.**

| **Study [Ref]** | **Type study** | **n** | **Setting** | **Period data** | **HBV women (HBsAg+)** | **Associations** | **Associated with HBV (risk and 95%CI)** | **Not associated** |
| --- | --- | --- | --- | --- | --- | --- | --- | --- |
| Wan (14) | Case control | 1,728 1,497 | Wuhan, China, hospital based | 2008-2015 | HBV+ 1,728 HBV- 1,497 | PTB and maternal viral load in 2nd trimester | aOR 1.18 (1.01-1.39) for each log10 copy/ml increase |  |
|  |  |  |  |  |  | PIH | aOR 2.2 CI 1.30-3.73 |  |
|  |  |  |  |  |  | Fetal distress | aOR 1.40 CI 1.09-1.78 |  |
|  |  |  |  |  |  | CS | aOR 1.70 CI 1.45-1.99 |  |
|  |  |  |  |  |  | Macrosomia | aOR 1.68 CI 1.19-2.37 |  |
| Ma (15) | SR & MA | 22 studies (only PTB reports) | Worldwide | 1970-2015 | 6,141,146  (81% data USA, Sweden) | PTB | pOR 1.16 CI 1.04-1.29 HBsAg+ HBeAg- |  |
|  |  |  |  |  |  | PTB | pRR 1.21 CI 1.10-1.32 HBsAg+ HBeAg+ |  |
| Keramat (13) | SR & MA | 18 studies | Not stated | 1990-2015 | number not stated | Nil |  | PROM, APH, SB, PIH, Pre-EC, EC |
| Cui (12) | Cohort | 21,004 in 1^st^ trimester | Jiangsu, China | 2012-2015 | HBV 513 (2.4%) | Miscarriage | aOR 1.71CI 1.23–2.38 | PTB, PPROM, GDM, ICP, LBW, SGA |
| Luo (11) | MA | 18 studies | Asia 15, Nth Am 2, Europe 2 | 2003-2012 | HBV 7,600 | PTB | OR 2.36 CI 1.81-3.09 | PND, EGA, SGA,LGA, BW, LBW, NJ, CA, macrosomia, Apgar 1^min^ |
|  |  |  |  |  |  | Asphyxia | OR 1.80 CI 1.27-2.56 |  |
| Huang (10) | MA | 9,098 | China, USA, Thailand | 1999-2012 | praevia cases | Nil |  | Placenta praevia |
|  |  | 15,571 |  |  | abruption cases | Nil |  | Placenta abruption |

Abbreviations (alphabetic order): Antepartum hemorrhage APH, adjusted Odds Ratio aOR, birth weight BW, Caesarean Section CS, Cohort (large >20,000 population based), Congenital abnormality CA, Eclampsia EC, intrahepatic cholestasis of pregnancy ICP, large for gestational age (LGA), low birth weight LBW, Meta-analysis MA; Neonatal Jaundice-NJ, Odds ratio OR, pooled Odds Ratio pOR, pooled Risk Ratio pRR, Pre-eclampsia Pre-EC, Pregnancy induced hypertension (or gestational hypertension) PIH, Preterm birth PTB, Preterm rupture of membranous PROM, Small for gestational age SGA, Stillbirth SB, Systematic Review-SR

**S2 Table. Characteristics of all women screened for hepatitis B virus.**

|  | **Included** | **Not Included**  **(did not meet criteria)** | **P-value^1^** |
| --- | --- | --- | --- |
|  | n=11,025 | n=4,089 |  |
| **Confirmed HBsAg positive** | 687/11,025 (6.2) | 250/3,802 (6.6) | 0.464 |
| **Confirmed HBeAg positive** | 211/687 (30.7) | 89/250 (35.6) | 0.179 |
| **Age yrs, mean±SD (min-max)** | 26±7 (13-50) | 26±7 (14-48) | 0.883 |
| **Age<25 years** | 5,060/11,025 (45.9) | 1,866/4,088 (45.6) | 0.784 |
| **Gravidity median [IQR] (min-max)** | 2 [1-4] (1-19) | 2 [1-4] (1-16) | 0.791 |
| **Parity median [IQR] (min-max)** | 1 [0-2] (0-15) | 1 [0-2] (0-13) | 0.678 |
| **Primigravida** | 3,522/11,025, (31.9) | 1,360/4,089, (33.3) | 0.207 |
| **Karen heritage** | 7,162/11,025 (65.0) | 2,250/4,087 (55.1) | <0.001 |
| **Migrant (not refugee)** | 6,452/11,025 (58.5) | 3,137/4,089 (76.7) | <0.001 |
| **Literate** | 6,728/11,025 (61.0) | 2,414/4,087 (59.1) | 0.029 |
| **Smoker** | 1,440/11,025 (13.1) | 561/4,086 (13.7) | 0.281 |
| **First ANC visit in 1st trimester** | 4,479/11,025 (40.6) | 1,443/4,084 (35.3) | <0.001 |
| **Underweight, n (%) (BMI<18.5 kg/m2)** | 1,060/10,987 (9.6) | 444/4,029 (11.0) | 0.004 |
| **Height (cm) mean (min-max)** | 151±5 (130-191) | 152±5 (120-169) | 0.143 |
| **Trimester of last ANC visit** |  | 3,549/4,089 (86.8) |  |
| **First trimester** |  | 451/3,549 (12.7) |  |
| **Second trimester** |  | 1,064/3,549 (30.0) |  |
| **Third trimester** |  | 2,034 (57.3) |  |
| **Previous abortion (if gravidity >1),** | 2,321/7,503, (30.9) | 856/2,676, (32.0) | 0.312 |
| **Previous preterm birth (if gravidity>1)** | 589/7,503, (7.9) | 222/2,672 (8.3) | 0.453 |
| Data are n (%) unless otherwise stated  ^1^ p value of 0.05 is considered statistically significant | |  |  |

**S3. Table Baseline characteristics of pregnant women with HBsAg+ confirmed (included) and HBsAg+ unconfirmed (excluded) status**

|  | **HBsAg+ confirmed**  **(included women)** | **HBsAg+ unconfirmed (excluded women)** | **P-value^1^** |
| --- | --- | --- | --- |
|  | n=687 | n=469 |  |
| **Age yrs, mean±SD (min-max)** | 27±7 (15-47) | 27±7 (14-47) | 0.485 |
| **Age<25 years** | 278/687 (40.5) | 188/469 (40.1) | 0.903 |
| **Gravidity median [IQR]** | 3 [1-4] | 2 [1-4] | 0.781 |
| **Parity median [IQR]** | 1 [1-3] | 1[0-3] | 0.830 |
| **Primigravida** | 173/687 (25.2) | 126/469 (26.9) | 0.539 |
| **Karen ethnicity** | 495/687 (72.1) | 325/469 (69.3) | 0.323 |
| **Status Migrant (not refugee)** | 389/687 (56.6) | 295/469 (62.9) | 0.033 |
| **Migrant: lives in Myanmar** | 219/386 (56.7) | 157/280 (56.1) | 0.874 |
| **Visitor to Refugee camp** | 44/687 (6.4) | 41/469 (8.7) | 0.138 |
| **Literate** | 398/687 (57.9) | 268/469 (57.1) | 0.809 |
| **Smoker** | 102/687 (14.8) | 70/469 (14.9) | 1.000 |
| **First ANC visit in 1st trimester** | 289/687 (42.1) | 159/469 (34.0) | 0.007 |
| **Underweight^2^, (BMI<18.5 kg/m^2^)** | 52/684 (7.6) | 37/464 (8.0) | 0.823 |
| **Overweight^2^, (BMI≥23 kg/m^2^)** | 279/684 (40.8) | 177/464 (38.1) | 0.668 |
| **Previous miscarriage (Gravidity >1)** | 150/514 (29.2) | 106/343 (30.9) | 0.595 |
| **Previous PTB (Gravidity>1)** | 48/514 (9.3) | 26/343 (7.6) | 0.388 |

Data are n (%), mean±standard deviation (SD) (min-max); median inter-quartile range [IQR] (min-max), Abbreviations: ANC antenatal clinic, BMI body mass index, PTB preterm birth

^1^ p value of 0.05 is considered statistically significant

^2^ BMI if trimester at first ANC was trimester one, as a proxy for pre pregnancy weight.

**S4. Table Pregnancy outcomes of pregnant women with HBsAg+ confirmed (included) and HBsAg+ unconfirmed (excluded) status**

| \|  \| **HBsAg+ confirmed**  **(included women)** \| **HBsAg+ unconfirmed (excluded women)** \| **P-value^1^** \| \| --- \| --- \| --- \| --- \| \|  \| n=687 \| n=469 \|  \| \| **Miscarriage (1st ANC Trimester 1)** \| 63/289 (21.8%) \| 12/65 (18.5%) \| 0.617 \| \| **Delivered (known outcome)** \| n=607 \| n=166 \|  \| \| **Gestation, weeks mean±SD (min-max)** \| 39.1 ±1.6 (28.1-43.2) \| 38.8 ±2.0 (29.6-42.1) \| 0.131 \| \| **Preterm birth, (<37 wks)** \| 34/607 (5.6%) \| 15/156 (9.6%) \| 0.097 \| \| **Stillbirth** \| 5/607 (0.8%) \| 3/166 (1.8%) \| Too few \| \| **Congenital abnormality** \| 9/606 (1.5%) \| 2/165 (1.2%) \| 1.000 \| \| **If liveborn, normal singleton** \| 593 \| 160 \|  \| \| **Weighed in 72 hrs of birth** \| 547/593 (92.2%) \| 155/160 (96.9%) \| 0.049 \| \| **Birthweight, grams mean±SD (min-max)** \| 3,067±483 (1,000-5,700) \| 3,018±524 (1,070-4,295) \| 0.272 \| \| **Small for gestational age** \| 84/544 (15.4%) \| 29/154 (18.8%) \| 0.323 \| \| **Delivered by Caesarean section** \| 47/607 (7.7%) \| 16/166 (9.6%) \| 0.425 \| \| **Neonatal death** \| 3/592 (0.5%) \| 1/160 (0.6%) \| Too few \| |
| --- | --- | --- | --- | --- | --- | --- | --- | --- | --- | --- | --- | --- | --- | --- | --- | --- | --- | --- | --- | --- | --- | --- | --- | --- | --- | --- | --- | --- | --- | --- | --- | --- | --- | --- | --- | --- | --- | --- | --- | --- | --- | --- | --- | --- | --- | --- | --- | --- | --- | --- | --- | --- | --- | --- | --- | --- |

Data are n (%) unless otherwise stated

^1^ p value of 0.05 is considered statistically significant

**S5. Table Baseline characteristics of the pregnant women with HBeAg+ confirmed who were included or excluded**

|  | **HbeAg+**  **(included women)** | **HbeAg+**  **(excluded women)** | **P-value^1^** |
| --- | --- | --- | --- |
|  | n=211 | n=89 |  |
| **Age yrs, mean±SD (min-max)** | 23±6 (15-45) | 25±6 (15-45) | 0.033 |
| **Age<25 years** | 144/211 (68.2) | 48/89 (53.9) | 0.025 |
| **Gravidity median [IQR]** | 2 [1-3] | 2 [1-3] | 0.157 |
| **Parity median [IQR]** | 1 [0-2] | 1 [0-2] | 0.127 |
| **Primigravida** | 83/211 (39.3) | 33/89 (37.1) | 0.795 |
| **Karen ethnicity** | 154/211 (73.0) | 52/89 (58.4) | 0.015 |
| **Status Migrant (not refugee)** | 140/211 (66.4) | 64/89 (71.9) | 0.416 |
| **Literate** | 124/211 (58.8) | 53/89 (59.6) | 1.000 |
| **Smoker** | 22/211 (10.4) | 16/89 (18.0) | 0.087 |
| **First ANC visit in 1st trimester** | 83/211 (39.3) | 26/89 (29.2) | 0.246 |
| **Underweight^2^, (BMI<18.5 kg/m^2^)** | 16/210 (7.6) | 8/89 (9.0) | 0.650 |
| **Overweight^2^, (BMI≥23 kg/m^2^)** | 62/210 (29.5) | 34/89 (38.2) | 0.264 |
| **Previous miscarriage (Gravidity >1)** | 36/128 (28.2) | 15/56 (26.8) | 1.000 |
| **Previous PTB (Gravidity>1)** | 15/128 (11.7) | 6/56 (10.7) | 1.000 |

Data are n (%), mean±standard deviation (SD) (min-max); median inter-quartile range [IQR] (min-max), Abbreviations: ANC antenatal clinic, BMI body mass index, PTB preterm birth

^1^ p value of 0.05 is considered statistically significant

^2^ BMI if trimester at first ANC was trimester one, as a proxy for pre pregnancy weight;
